# Wearable Sports Vision Training May Improve Selected Visuomotor Outcomes and Hitting Precision in Collegiate Badminton Athletes: A Randomized Controlled Trial

**DOI:** 10.3390/diagnostics16121769

**Published:** 2026-06-08

**Authors:** Yun-Wei Chiang, Jia-Yuan Chang, Chi-Hung Lee, Ching-Wen Huang, Shou-Chun Wei, Shang-Min Yeh, Shuan-Yu Huang, Wei-Chin Hung, Yuh-Ling Shyu

**Affiliations:** 1 Department of Optometry, Central Taiwan University of Science and Technology, Taichung 406, Taiwan; 108589@ctust.edu.tw (Y.-W.C.); 107489@ctust.edu.tw (S.-M.Y.); 108695@ctust.edu.tw (S.-Y.H.); 2 Program of Electrical and Communications Engineering, Feng Chia University, Taichung 407, Taiwan; 3 Department of Optometry, University of Kang Ning, Taipei 114, Taiwan; 4 Department of Electrical Engineering, Feng Chia University, Taichung 407, Taiwan; chihlee@fcu.edu.tw; 5 Department of Ophthalmology, Fu Jen Catholic University Hospital, New Taipei City 242, Taiwan; ophcwh@gmail.com; 6 Department of Athletics, Central Taiwan University of Science and Technology, Taichung 406, Taiwan; scwei@ctust.edu.tw; 7 Department of Physics, R.O.C. Military Academy, Kaohsiung 830, Taiwan

**Keywords:** wearable sports technology, sports vision training, visuomotor performance, reaction performance, hitting precision, badminton athletes

## Abstract

**Background**: High-level badminton performance requires rapid perceptual processing, visuomotor coordination, and precise movement responses under continuously changing spatial conditions. Although wearable sports vision interventions have shown potential for enhancing perceptual–motor performance, evidence regarding their longitudinal effects and transfer to sport-specific outcomes remains limited. **Trial design**: A single-center, exploratory randomized controlled trial using a parallel-group structure. Simple randomization without blocking or stratification resulted in a final allocation ratio of 16:10 (approximately 1.6:1) between the training and control groups. **Methods**: Twenty-six collegiate badminton athletes aged 18–25 were randomized into a wearable sports vision training group (n = 16) or a control group (n = 10). The intervention group completed wearable sports vision training using Automatic Dual Rotational Risley Prisms (ADRRPs) for 15 min twice weekly over 4 weeks. **Results**: Baseline-adjusted ANCOVA demonstrated significant between-group effects for reaction time (*p* = 0.003) and target-zone accurate hits (*p* = 0.004), whereas binocular visual function outcomes did not show statistically significant between-group differences. No adverse events were reported. **Conclusions**: Four weeks of wearable sports vision training may be associated with improvements in selected visuomotor outcomes, particularly reaction performance and target-zone hitting accuracy, in collegiate badminton players. Larger trials are needed to evaluate long-term retention and broader sport-specific applicability. **Trial registration**: ClinicalTrials.gov Identifier: NCT07105462, registered 29 July 2025.

## 1. Background

Badminton is a visually demanding racket sport requiring rapid perceptual processing, anticipatory decision-making, and precise visuomotor coordination under dynamic spatial conditions. Athletes must continuously respond to unpredictable shuttlecock trajectories while maintaining accurate movement timing and stroke execution. Consequently, perceptual–motor efficiency and sensorimotor responsiveness are increasingly recognized as important contributors to competitive athletic performance [1,2].

Sport vision training (SVT) has therefore gained increasing attention as a strategy for enhancing perceptual and motor abilities in athletes. Evidence from interceptive sports suggests that targeted visual training may improve gaze control, tracking efficiency, visuomotor timing, and decision-making under pressure [3,4]. Within this context, the vergence system is particularly relevant because it supports clear and single binocular vision during repeated shifts of focus between moving objects and near-contact zones. Previous work has also suggested that efficient vergence function may contribute to visuomotor readiness in high-speed sporting environments [5,6].

Emerging evidence indicates that vergence-focused visual exercises may influence selected aspects of oculomotor function [7,8,9]. However, existing evidence regarding wearable sports vision interventions remains limited, particularly in relation to longitudinal adaptation and transfer to sport-specific performance outcomes.

Accordingly, the present randomized controlled trial investigated whether a four-week wearable sports vision intervention program could improve visuomotor responsiveness and badminton-specific performance outcomes in collegiate athletes.

## 2. Methods

### 2.1. Subjects

Collegiate badminton players aged 18 to 25 years were recruited from Central Taiwan University of Science and Technology and National Taichung University of Education. The study protocol was reviewed and approved by the Human Research Ethics Committee of China Medical University Hospital, Taichung, Taiwan (CRREC-114-011), and all procedures adhered to the principles of the Declaration of Helsinki. Written informed consent was obtained from all participants prior to study enrollment. Recruitment occurred between March and April 2025, and all post-intervention assessments were completed by May 2025.

Participants were eligible if they were between 18 and 25 years of age and had participated in organized badminton training for at least two years. Individuals with ocular, neurological, or systemic conditions potentially affecting visual or visuomotor performance were excluded.

As this was an exploratory randomized controlled trial, a formal sample size calculation was not performed. A convenience sample of 26 players was enrolled and randomly allocated to the vergence training group (n = 16) or the control group (n = 10). In this exploratory pilot trial, recruitment of collegiate badminton athletes was challenging because of training and competition schedules. Therefore, the study primarily aimed to obtain preliminary data regarding wearable sports vision training. Because this exploratory pilot design focused on feasibility and hypothesis generation, a simple randomization approach was selected to maintain procedural feasibility and avoid introducing additional stratification factors in a relatively small sample. Simple randomization without blocking or stratification was used, and the resulting 16:10 allocation ratio occurred during the random allocation process.

### 2.2. Experimental Design

This exploratory randomized controlled trial investigated whether a four-week wearable sports vision intervention could produce measurable changes in visuomotor responsiveness and badminton-specific performance in collegiate players.

**Figure 1:**
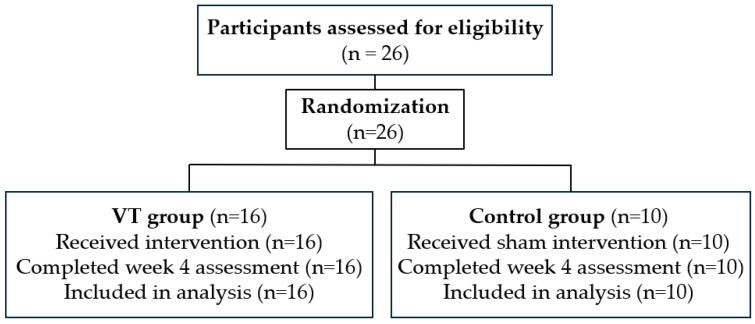
CONSORT flow diagram of participant enrollment, randomization, allocation, follow-up, and analysis.

A parallel-group design was implemented with an allocation ratio of approximately 1.6:1. An independent researcher generated the randomization sequence using the RAND function in Microsoft Excel (Microsoft Corp., Redmond, WA, USA), applying simple randomization without stratification or blocking. Allocation concealment was maintained with sequentially numbered, opaque, sealed envelopes, which were opened only after each participant had been enrolled. The individual responsible for recruitment did not have access to the allocation sequence, ensuring proper concealment throughout the assignment process. Personnel enrolling participants and delivering the intervention were not involved in sequence generation. Participant enrollment, randomization, allocation, intervention completion, and final analysis are summarized in Figure 1.

To maintain participant blinding, both the intervention and control groups used ADRRPs headsets that were identical in appearance. The training group received wearable sports vision sessions produced by rotating prisms, whereas the control group used headsets fitted with plano lenses, creating a sham condition that mimicked the look and feel of the device without introducing vergence demands. Outcome assessors and data analysts were not blinded to group allocation.

Participants in the training group completed ADRRPs-based vergence exercises for 15 min per session, twice weekly, for a total of four weeks. The prism stimulus was individualized according to each participant’s positive fusional vergence (PFV) breakpoint to determine an appropriate range of vergence challenge. Control participants wore visually identical ADRRPs headsets fitted with plano lenses under the same supervised indoor training conditions as the intervention group. Participants remained visually engaged by performing the same fixation and visual tracking tasks throughout the 15-min sessions, but without prism-induced vergence stimulation.

**Figure 2:**
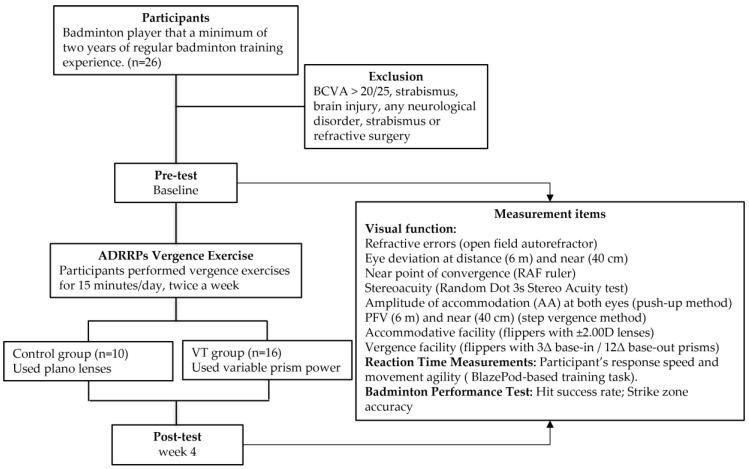
Overview of the experimental protocol and assessment procedures. Participants completed baseline assessments (week 0), followed by a four-week intervention period consisting of ADRRPs-based vergence training or sham-control sessions, and post-intervention assessments at week 4. Visual function assessments, reaction time measurements, and badminton performance tests were conducted at both time points.

Assessments of binocular visual function, visuomotor reaction time, and badminton-specific hitting performance were administered at baseline (week 0) and after the intervention period (week 4). Figure 2 provides an overview of the experimental protocol and assessment procedures. All intervention sessions were supervised by a certified optometrist. No eligibility criteria were imposed for interventionists or study sites. Given the exploratory nature, relatively small sample size, and short duration of the study, no interim analyses or stopping rules were defined.

Primary outcomes were vergence facility, accommodative facility, reaction time, and target-zone accurate hits. Secondary outcomes included positive fusional vergence, near point of convergence, amplitude of accommodation, and successful hits.

### 2.3. Visual Function Assessments

Visual performance was evaluated using a standardized clinical assessment battery including refractive status, visual acuity, stereoacuity, accommodative performance, vergence-related function, and near visual measures. Assessments were performed under standardized clinical conditions by trained examiners using established optometric procedures. Outcome measures included accommodative facility, vergence facility, positive fusional vergence, near point of convergence, and amplitude of accommodation.

### 2.4. Reaction Time Measurements

**Figure 3:**
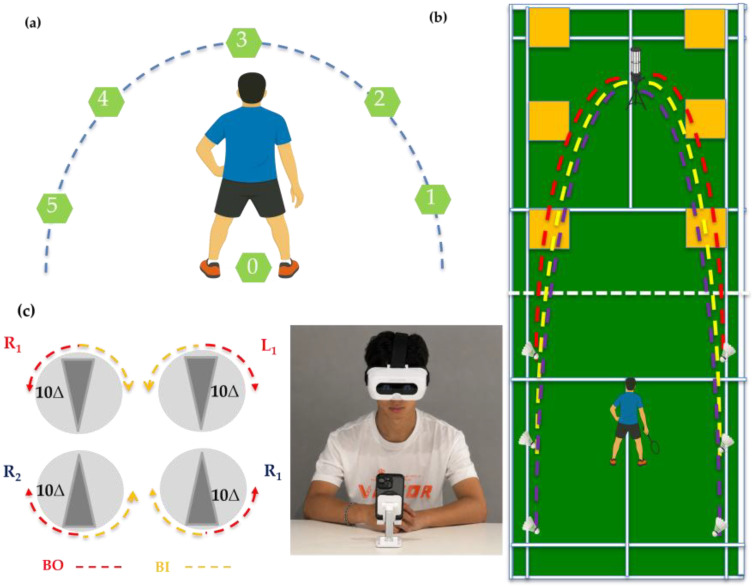
Experimental setup for the visuomotor reaction task, badminton performance assessment, and ADRRPs-based training. (**a**) Spatial arrangement of the light-response task; (**b**) badminton target zones used for evaluating hitting precision; and (**c**) internal configuration of the Risley prisms within the ADRRPs system and a player performing wearable sports vision training.

Reaction performance was assessed using a validated light-response system involving rapid upper-limb responses to randomized visual stimuli under dynamic conditions, as illustrated in Figure 3a. Participants completed repeated response trials, and mean reaction time was automatically recorded for analysis. Detailed configuration of the reaction task has been described previously.

### 2.5. Badminton Performance Test

The badminton performance test was used to assess the participants’ on-court reaction ability and shot accuracy under dynamic conditions. At the start of the test, each participant took a position at the center of the court in a ready stance. A shuttlecock launcher (MSI-AS1083 badminton shuttle machine; Ming Shiang Technology Co., Ltd., New Taipei City, Taiwan) was placed at the center of the opposite court and programmed with five selectable random modes.

In each test trial, the machine continuously launched 16 shuttlecocks toward six pre-defined landing zones, as illustrated in Figure 3b. These landing zones were strategically placed throughout the court to assess the participants’ reaction, movement, and shot accuracy across various areas. Six target zones were designated as follows:

(1) Two frontcourt positions—left and right drop shots (represented by red trajectories);
(2) Two midcourt positions—left and right flat drives (represented by yellow trajectories);
(3) Two backcourt positions—left and right flat clears (represented by purple trajectories).

For each trial, the machine randomly selected one of five preset modes. The sequence of shuttlecock trajectories and landing points was randomized within each trial, with landing zones receiving an average of 2–3 shuttlecocks each.

Participants were instructed to react to each launched shuttlecock and return it to the valid singles court area. After each return, participants were required to return to the center of the court and prepare for the next shuttlecock. The launcher delivered 16 shuttlecocks in approximately 21 s. Two primary performance indicators were recorded during the badminton performance test: (1) successful hits, indicating the total number of shuttlecocks successfully returned to the valid singles court area; (2) target-zone accurate hits, representing the number of shuttlecocks accurately returned to the designated target zones located in the frontcourt (left and right), midcourt (left and right), and backcourt (left and right), as shown in Figure 3b. Each target zone was defined as a square area measuring 100 cm × 100 cm (yellow squares in Figure 3b).

### 2.6. Visual Training with Automatic Dual Rotational Risley Prisms (ADRRPs)

Participants in the intervention group underwent individualized wearable sports vision training using the ADRRPs system under supervised conditions, as illustrated in Figure 3c. The intervention delivered repeated dynamic convergence–divergence stimulation based on each participant’s vergence capability during near visual tasks. Training sessions lasted 15 min and were conducted twice weekly for four weeks. Detailed technical specifications of the ADRRPs system and training configuration have been reported previously.

### 2.7. Statistics

All statistical analyses were conducted using IBM SPSS Statistics for Windows, Version 19.0 (IBM Corp., Armonk, NY, USA). The normality of continuous variables was assessed using the Shapiro–Wilk test. Descriptive data are presented as mean ± standard deviation for consistency and ease of interpretation. Within-group comparisons between baseline and week 4 were performed using paired-sample *t*-tests for normally distributed outcomes (vergence facility, accommodative facility, and reaction time) or Wilcoxon signed-rank tests for non-normally distributed or count-based outcomes (positive fusional vergence, near point of convergence, amplitude of accommodation, successful hits, and target-zone accurate hits). In accordance with the statistical plan, only one within-group test was applied to each variable based on its distributional characteristics.

To further adjust for baseline differences, analysis of covariance (ANCOVA) was conducted with baseline values included as covariates. A two-tailed *p*-value < 0.05 was considered statistically significant. Given the exploratory nature of this pilot trial and the limited sample size, adjustments for multiple comparisons were not applied. Accordingly, statistically significant findings should be interpreted as preliminary and hypothesis-generating rather than confirmatory. All randomized participants completed both baseline and post-intervention assessments and were included in the final analyses; therefore, no imputation for missing data was required.

## 3. Results

### 3.1. Participant Characteristics

Baseline demographic and screening characteristics are summarized in Table 1. No statistically significant between-group differences were observed at baseline in age, refractive status, visual acuity, or stereoacuity. However, moderate numerical differences in selected visual characteristics were present and should be interpreted cautiously given the limited sample size of this exploratory trial. Baseline values for the primary and secondary visual function outcomes are presented in Table 2. All 26 randomized participants completed the intervention and post-intervention assessments and were included in the final analysis.

#### Changes in Visual Performance Measures After the 4-Week Intervention

Changes in visual performance measures across the four-week intervention are summarized in Table 2. Although within-group improvements in vergence facility (VF) and accommodative facility (AF) were observed in the training group, baseline-adjusted ANCOVA analyses demonstrated no statistically significant between-group effects for either outcome. In contrast, positive fusional vergence (PFV), near point of convergence (NPC), and amplitude of accommodation (AA) remained relatively stable throughout the intervention period, with no significant within-group or between-group differences observed after baseline adjustment. Overall, binocular visual function outcomes did not demonstrate statistically significant between-group differences and should therefore be interpreted as exploratory and hypothesis-generating.

### 3.2. Changes in Reaction Time Performance

**Figure 4:**
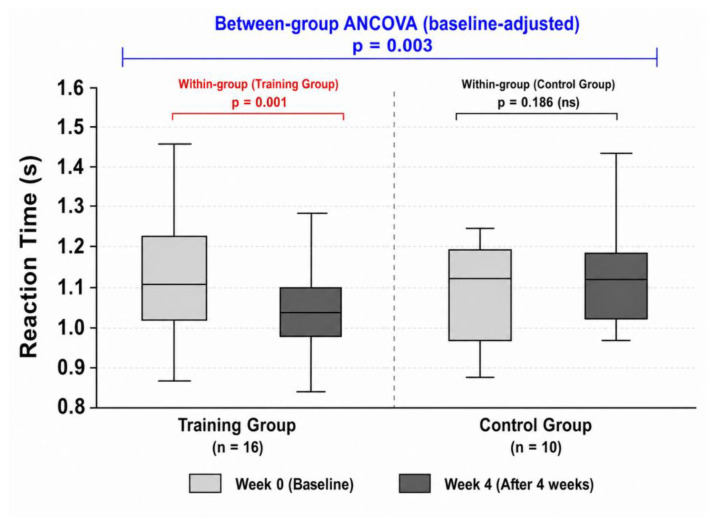
Boxplots of reaction time (RT) at baseline (Week 0) and after the four-week intervention (Week 4) for the training and control groups. Boxes represent the interquartile range (IQR), the median is shown by the horizontal line, and whiskers extend to 1.5 × IQR. Within-group *p*-values are shown above each group comparison, whereas the upper horizontal line indicates the baseline-adjusted between-group ANCOVA result.

Figure 4 illustrates reaction time (RT) values for both groups at baseline and week 4. Baseline-adjusted ANCOVA demonstrated a significant between-group effect for reaction time (*p* = 0.003).

### 3.3. Changes in Successful Hits

Successful hits showed small and non-significant changes in both groups over the four-week intervention period. In the training group, the mean number of successful hits increased slightly from 9.94 ± 2.10 at baseline to 10.50 ± 2.32 at week 4 (Wilcoxon signed-rank test, *p* = 0.544). In the control group, successful hits decreased modestly from 7.90 ± 2.45 to 7.00 ± 2.10 (Wilcoxon signed-rank test, *p* = 0.240). ANCOVA adjusted for baseline values demonstrated no significant between-group effect for successful hits (*p* = 0.22).

### 3.4. Changes in Target-Zone Accurate Hits

**Figure 5:**
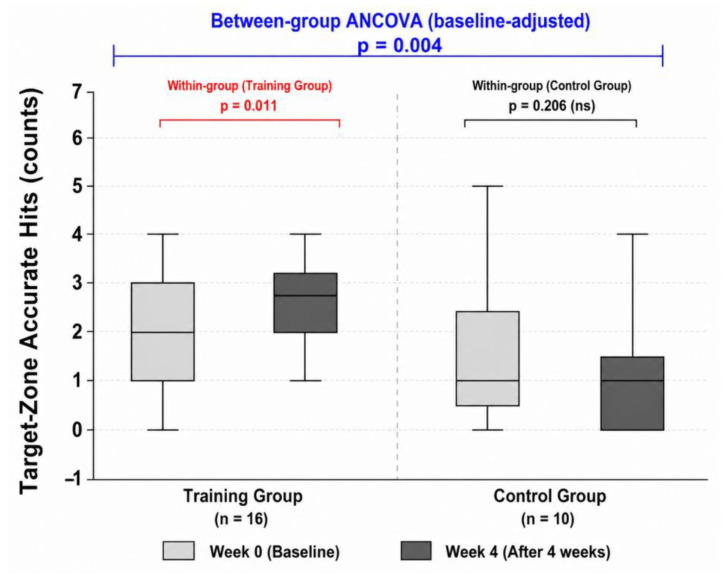
Boxplots of target-zone accurate hits at baseline (Week 0) and after the four-week intervention (Week 4) for both groups. Boxes represent the interquartile range (IQR), the median is indicated by the horizontal line, and whiskers extend to 1.5 × IQR. Within-group *p*-values are shown above each group comparison, whereas the upper horizontal line indicates the baseline-adjusted between-group ANCOVA result.

Figure 5 illustrates changes in target-zone accurate hits between baseline and week 4. The training group increased from 2.21 ± 1.18 to 2.95 ± 1.31 hits, whereas the control group decreased from 1.73 ± 1.90 to 1.18 ± 1.60 hits. Baseline-adjusted ANCOVA demonstrated a significant between-group effect for target-zone accurate hits (*p* = 0.004).

## 4. Discussion

The present randomized controlled trial investigated whether a four-week wearable sports vision intervention could induce measurable adaptations in visuomotor responsiveness and badminton-specific performance in collegiate athletes. The findings suggest that wearable sports vision training may be associated with improvements in selected visuomotor outcomes, particularly reaction performance and hitting precision, whereas traditional binocular visual function measures remained statistically unchanged compared with the control group after baseline-adjusted analyses.

Across the intervention period, the most consistent intervention-related effects were observed in reaction performance and target-zone hitting accuracy, whereas traditional binocular visual function measures did not demonstrate statistically significant between-group differences after baseline adjustment. In high-speed racket sports such as badminton, even relatively small reductions in visuomotor reaction time may contribute to improved interception timing, defensive responsiveness, and rapid decision-making during fast-paced rallies. Similarly, improved target-zone hitting accuracy may reflect enhanced visuomotor coordination and shuttle placement precision, which could potentially contribute to tactical advantages during competitive play. However, the minimal clinically or competitively meaningful changes for these measures in badminton athletes remain unclear and warrant further investigation in larger longitudinal studies.

These findings are generally aligned with previous literature suggesting that sports vision interventions may enhance perceptual–motor responsiveness and reaction efficiency in athletes participating in visually demanding sports [2,3,10,11]. Previous studies have also reported that visual–motor training may contribute to improved coordination, attentional processing, and sport-specific performance under dynamic competitive conditions [4,5,12,13].

However, the relationship between improvements in visual function and real-world sports-specific performance remains complex. As reported by Poltavski et al. [14], enhancements in visual processing and reaction speed may not automatically translate into refined technical execution unless accompanied by sport-specific cognitive or motor training [14]. Rezaee et al. also demonstrated that integrated visual–motor programs may outperform vision-only interventions in improving athletic performance [13], suggesting that the pathway from sensory improvement to skilled motor output may require multimodal reinforcement.

Collectively, the present findings should be considered exploratory and hypothesis-generating. Larger adequately powered trials are required to confirm whether the observed improvements in reaction performance and target-zone accuracy translate into meaningful sport-specific performance benefits.

### Limitations

This trial has several limitations that should be acknowledged. First, the sample size was relatively small, and participants were recruited from a limited athletic population within a single geographic region, which may reduce the generalizability of the findings. Because simple randomization was used in a relatively small exploratory sample, unequal group allocation and potential baseline imbalance cannot be excluded. Although no statistically significant between-group differences in refractive status were detected at baseline, moderate numerical differences were observed between groups, and their potential influence on visuomotor adaptation cannot be completely excluded. Variations in refractive status may affect retinal image quality, accommodative demand, and binocular visual processing, which could potentially influence reaction performance and hitting precision during dynamic sports tasks. Future confirmatory trials should consider block or stratified randomization procedures, including stratification based on refractive status, to improve group balance, reduce potential confounding effects, and enhance methodological rigor. Second, because only pre- and post-intervention assessments were performed, the temporal progression of training-related adaptations across the intervention period could not be determined. Third, the intervention lasted only four weeks, which may have been insufficient to produce measurable changes in certain traditional binocular outcomes or stable physiological adaptations in oculomotor mechanisms. Therefore, the observed improvements may reflect short-term functional adaptation rather than long-term physiological change. Potential adaptation or fatigue effects during repeated training sessions were not formally quantified in the present exploratory trial and warrant further investigation in future longitudinal studies. Future studies incorporating longer intervention periods and extended follow-up assessments are warranted to determine the persistence and clinical significance of these effects. In total, four primary outcomes (vergence facility, accommodative facility, reaction time, and target-zone accurate hits) and four secondary outcomes (positive fusional vergence, near point of convergence, amplitude of accommodation, and successful hits) were evaluated without formal multiplicity adjustment. Therefore, the possibility of Type I error cannot be excluded. Accordingly, the statistically significant findings should be interpreted cautiously until validated in larger confirmatory trials with prespecified primary endpoints. Another limitation is the absence of assessor and analyst blinding, which may have introduced potential detection or analytical bias, particularly for examiner-dependent assessments such as vergence facility, accommodative facility using flipper lenses, and near point of convergence measured with the push-up method. These procedures involve subjective participant responses and examiner judgment, which may have influenced the recorded outcomes. In addition, performance-based outcomes such as reaction time measurements and badminton target-zone assessments may also have been influenced by nonspecific expectancy or motivational effects despite the use of sham-control masking. Future confirmatory trials incorporating blinded outcome assessment and standardized data-processing procedures are warranted to further strengthen methodological rigor. In addition, recruiting larger and more diverse athlete populations and comparing different training dosages or combined visual–motor interventions may help clarify the broader applicability and practical utility of wearable sports vision training. The present findings should be viewed primarily as exploratory evidence intended to inform the design of future adequately powered confirmatory trials.

## 5. Conclusions

This exploratory randomized controlled trial provides preliminary evidence regarding the feasibility of wearable sports vision training in collegiate badminton athletes. Wearable sports vision training may be associated with improvements in selected visuomotor outcomes, particularly reaction performance and hitting precision, while effects on binocular visual function remain inconclusive and require further investigation in larger trials. Future studies with larger sample sizes, longer intervention periods, and blinded assessments are warranted to further clarify the clinical and sport-specific relevance of these findings.

## Figures and Tables

**Table 1:** Baseline Comparison of Demographic and Visual Function Characteristics Between Control and Visual Training Groups.

	Control	VT	*p*
**N**	10	16	
**Age**	18.79 ±1.12	19.45 ± 1.28	0.200
**OD SE (D)**	−1.02 ± 1.24	−2.21 ± 1.79	0.083
**OS SE (D)**	−1.15± 1.31	−2.24 ± 1.79	0.113
**OD VA (logMAR)**	−0.14 ± 0.11	−0.10 ± 0.08	0.346
**OS VA (logMAR)**	−0.16 ± 0.08	−0.09 ± 0.09	0.052
**Stereoacuity (sec)**	26.5 ± 10.55	26.3 ± 9.53	0.968

Data are presented as mean ± standard deviation. Baseline comparisons were performed using independent-samples tests as appropriate. OD: right eye; OS: left eye; SE: spherical equivalent; VA: visual acuity.

**Table 2:** Changes in visual performance measures after the 4-week intervention.

Outcome	Group	Baseline (Mean ± SD)	Week 4 (Mean ± SD)	Within-Group Test, *p*-Value	ANCOVA (*p*)
**Vergence facility (cpm)**	Training	20.93 ± 6.02	25.17 ± 6.68	Paired *t*-test, ***p*** = **0.001**	0.15
	Control	22.00 ± 4.05	23.75 ± 5.02	Paired *t*-test, *p* = 0.140	—
**Positive fusional vergence (Δ)**	Training	26.67 ± 7.92	29.07 ± 6.18	Wilcoxon signed-rank, *p* = 0.178	0.41
	Control	22.40 ± 7.04	22.80 ± 8.65	Wilcoxon signed-rank, *p* = 0.605	—
**Near point of convergence (cm)**	Training	6.90 ± 2.90	6.74 ± 2.84	Wilcoxon signed-rank, *p* = 0.301	0.43
	Control	6.09 ± 1.12	5.84 ± 1.43	Wilcoxon signed-rank, *p* = 0.275	—
**Accommodative facility (cpm)**	Training	18.53 ± 4.19	21.56 ± 5.38	Paired *t*-test, ***p*** = **0.021**	0.07
	Control	18.30 ± 3.74	19.25 ± 3.85	Paired *t*-test, *p* = 0.085	—
**Amplitude of accommodation (D)**	Training	10.85 ± 1.41	10.60 ± 1.13	Wilcoxon signed-rank, *p* = 0.303	0.29
	Control	10.76 ± 1.15	11.21 ± 1.00	Wilcoxon signed-rank, *p* = 0.103	—

Within-group tests were selected based on Shapiro–Wilk normality assessment: paired *t*-tests for normally distributed variables and Wilcoxon signed-rank tests for non-normal distributions. ANCOVA models further adjusted for baseline values.

## Data Availability

The datasets generated and analyzed during the current study are available from the corresponding author upon reasonable request.

## Supplementary Materials

The following supporting information can be downloaded at: https://www.mdpi.com/article/10.3390/diagnostics16121769/s1, Table S1: CONSORT 2025 checklist item description.

## List of Abbreviations

**Abbreviation**	**Definition**
VT	Visual Training
ADRRPs	Automatic Dual Rotational Risley Prisms
VF	Vergence Facility
AF	Accommodative Facility
NPC	Near Point of Convergence
PFV	Positive Fusional Vergence
SVT	Sport Vision Training
AA	Amplitude of Accommodation
OD	The Right Eye
OS	The Left Eye
SE	The Spherical Equivalent Refractive Error
